# The Impact of Steroid Activation of TRPM3 on Spontaneous Activity in the Developing Retina

**DOI:** 10.1523/ENEURO.0175-19.2020

**Published:** 2020-04-10

**Authors:** Corey M. Webster, Joshua Tworig, Franklin Caval-Holme, Catherine W. Morgans, Marla B. Feller

**Affiliations:** 1 Department of Molecular and Cell Biology, University of California. Berkeley, Berkeley, CA 94720-3200; 2 Helen Wills Neuroscience Institute, University of California, Berkeley, Berkeley, CA 94720-3200; 3 Department of Physiology and Pharmacology, Oregon Health and Science University, Portland, OR 97239

**Keywords:** Müller glia, retinal ganglion cell, retinal wave, two-photon calcium imaging

## Abstract

In the central nervous system, melastatin transient receptor potential (TRPM) channels function as receptors for the neurosteroid pregnenolone sulfate (PregS). The expression and function of TRPM3 has been explored in adult retina, although its role during development is unknown. We found, during the second postnatal week in mice, TRPM3 immunofluorescence labeled distinct subsets of inner retinal neurons, including a subset of retinal ganglion cells (RGCs), similar to what has been reported in the adult. Labeling for a TRPM3 promoter-driven reporter confirmed expression of the TRPM3 gene in RGCs and revealed additional expression in nearly all Müller glial cells. Using two-photon calcium imaging, we show that PregS and the synthetic TRPM3 agonist CIM0216 (CIM) induced prolonged calcium transients in RGCs, which were mostly absent in TRPM3 knock-out (KO) mice. These prolonged calcium transients were not associated with strong membrane depolarizations but induced c-Fos expression. To elucidate the impact of PregS-activation of TRPM3 on retinal circuits we took two sets of physiological measurements. First, PregS induced a robust increase in the frequency but not amplitude of spontaneous postsynaptic currents (PSCs). This increase was absent in the TRPM3 KO mice. Second, PregS induced a small increase in cell participation and duration of retinal waves, but this modulation persisted in TRPM3 KO mice, indicating PregS was acting on wave generating circuits independent of TRPM3 channels. Though baseline frequency of retinal waves was slightly reduced in the TRPM3 KO mice, other properties of waves were indistinguishable from wildtype. Together, these results indicate that the presence of neurosteroids impact spontaneous synaptic activity and retinal waves during development via both TRPM3-dependent and independent mechanisms.

## Significance Statement

Melastatin transient receptor potential (TRPM)3, a heat sensitive ion channel found throughout the CNS, also functions as a receptor for the neurosteroid pregnenolone sulfate (PregS). In the hippocampus and cerebellum, TRPM3 has been shown to have a functional role as a steroid receptor during development. We show that, in the developing retina, TRPM3 responds to PregS, producing prolonged calcium transients in a subset of retinal ganglion cells (RGCs) and increasing the frequency of spontaneous synaptic current onto RGCs. The PregS-mediated increase in spontaneous synaptic activity was absent in the TRPM3 knock-out (KO) retina. In addition, the absence of TRPM3 signaling reduced wave frequency. Thus, we show that TRPM3 and the endogenous neurosteroid, PregS, function in modulating spontaneous activity in the retina during development.

## Introduction

Melastatin transient receptor potential (TRPM) channels are polymodal cation conductances that are found throughout the body, including the peripheral nervous system where they play a key role in heat and pain sensation (Vriens et al., 2011; Vriens and Voets, 2018). TRPM3 is a cation channel with a high permeability to calcium (Grimm et al., 2003; Oberwinkler and Philipp, 2014), with the extent of permeability dependent on which splice isoforms are expressed (Oberwinkler et al., 2005). In addition to being activated by heat, TRPM3 is strongly activated by the neurosteroid pregnenolone sulfate (PregS; Wagner et al., 2008) as well as the synthetic small molecule CIM0216 (CIM; Held et al., 2015a). Activation of TRPM3 via PregS or CIM leads to a dramatic increase in intracellular calcium, which in turn strongly activates signaling cascades that alter gene transcription (Thiel et al., 2017).

The role of TRPM3 in the central nervous system is less well explored (Thiel et al., 2017). TRPM3 is present in the adult mouse retina, where it is expressed by retinal ganglion cells (RGCs) as well as cells in the inner nuclear layer (Brown et al., 2015), including Müller glial cells (Gilliam and Wensel, 2011; https://portals.broadinstitute.org/single_cell/study/SCP3/retinal-bipolar-neuron-drop-seq). TRPM3 knock-out (KO) mice have normal retinal structure and electroretinogram (ERG) responses but an attenuated pupillary light reflex (Hughes et al., 2012), suggesting a role for TRPM3, either directly or indirectly, in modulating light detection by the tissues of the eye while not distorting the ERG. Interestingly, TRPM3 may play a critical role during development. For example, in the developing cerebellum, TRPM3 is present at glutamatergic synapses, and activation of TRPM3 by PregS potentiates spontaneous synaptic transmission (Zamudio-Bulcock and Valenzuela, 2011), suggesting a functional role in developing circuits.

Although the endogenous ligands for TRPM3 are not yet fully determined (Thiel et al., 2017), the neurosteroid PregS is a strong candidate (Wagner et al., 2008). PregS is synthesized within the nervous system (Kimoto et al., 2001; Harteneck, 2013), including the retina (Cascio et al., 2015). Moreover, significantly lower concentrations of PregS are needed to activate TRPM3 in elevated temperatures (Vriens et al., 2011), indicating a synergism between ligand and temperature sensing that has been described in other channels such as TRPV1 (Neelands et al., 2008). In addition to activating TRPM3, PregS has excitatory effects on neural circuits by a variety of other mechanisms (Harteneck, 2013), including as a negative allosteric modulator of GABA-A receptors, as a positive allosteric modulator of NMDA receptors, and through interactions with AMPA, kainate (Yaghoubi et al., 1998), glycine (Wu et al., 1997), and nicotinic acetylcholine receptors (Smith et al., 2014). PregS-induced depolarization of isolated RGCs is absent in the TRPM3 KO (Brown et al., 2015), indicating that PregS is a specific agonist of TRPM3 in the retina. However, the impacts of PregS on RGCs and other cell types in the intact retina are unknown.

Here, we use the mouse retina to explore a role for PregS and TRPM3 signaling during development. Before eye opening and the maturation of vision, the retina exhibits retinal waves, a term used to describe spontaneously generated, correlated bursts of action potentials that spread across the retinal ganglion and inner nuclear cell layers (Wong, 1999; Blankenship and Feller, 2010). We use immunohistochemistry, two photon calcium imaging, and whole-cell voltage clamp recordings to determine the impact of TRPM3 agonists and genetic deletion on cellular calcium dynamics, spontaneous synaptic signaling, and retinal waves.

## Materials and Methods

### Mice

All experiments were performed on mice aged postnatal day (P)8 to P15 of either sex from C57BL/6 wild-type (WT; Harlan Laboratories) or TRPM3 KO (Vriens et al., 2011; NIH-1697: LexKO 380). Animal procedures were approved by the Institutional Animal Care and Use Committees and conformed to the National Institutes of Health *Guide for the Care and Use of Laboratory Animals*, the Public Health Service Policy, and the Society for Neuroscience Policy on the Use of Animals in Neuroscience Research.

### Immunohistochemistry

Animals were deeply anesthetized with isoflurane and decapitated. Eyes were dissected in freshly made ACSF at room temperature by cutting just behind the ora serrata, then removing the lens. Eyecups were fixed for 20 min in ice-cold 4% paraformaldehyde (PFA) in 0.1 M phosphate buffer, pH 7.4 (PB). The eyecups were washed in PB, then sunk in sequentially increasing concentrations of sucrose in PB (10%, 20%, 30% w/v) and immersed in 30% sucrose solution overnight at 4°C. The tissue was cryoembedded in OCT tissue-embedding compound over dry ice and methanol, and then sectioned perpendicular to the eye cup in 16-μm sections using a Cryostat. Sections were mounted on Super-Frost glass slides, air-dried, and then stored at −80°C or used immediately for staining.

**Figure 1.**
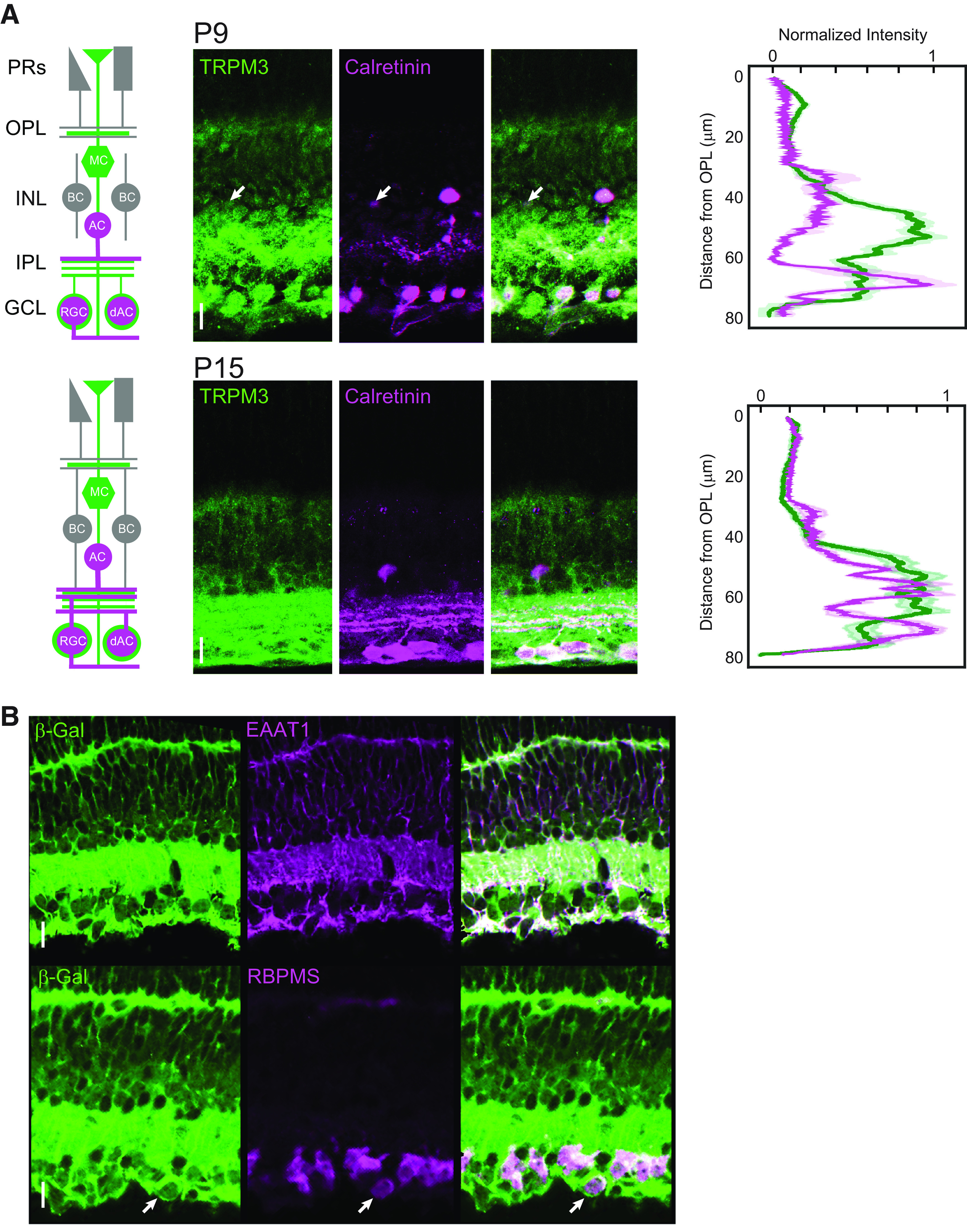
TRPM3 expression in postnatal retina. ***A***, left, Schematic of retinal circuit at P9 and P15. Middle, Immunofluorescence for TRPM3 and calretinin at two developmental ages. Calretinin is present in ACs, dACs, and some RGCs. White arrow, A TRPM3 immuno-positive putative AC. Right, Image intensity as a function of distance along the cross-section from the OPL for both TRPM3 (green) and calretinin (magenta). Bold lines are the average intensity from six ROIs and the light regions represent SD. Scale bar: 20 μm. ***B***, TRPM3 reporter co-localizes with Müller glia and RGCs. P12 TRPM3+/− retinas containing a β-Gal cassette in place of exon 17 of the coding sequence of TRPM3 were used to identify cells that express TRPM3 by immunofluorescence. Top, β-Gal co-localizes with EAAT1, a marker for Müller glia. Bottom, β-Gal is also present in the RGC layer, co-localizing with the RGC label, RBPMS (white arrow). PR: photoreceptors; MC: Müller cell; BC: bipolar cell; AC: amacrine cell; dAC: displaced amacrine cell; RGC: retinal ganglion cell; OPL: outerplexiform layer; INL: inner nuclear layer, IPL: inner plexiform layer.

Several antibodies were used. First, rabbit antiserum against amino acids 1543–1672 of mouse TRPM3 (accession AEE80504) was produced as previously described (Brown et al., 2015). For the anti-TRPM3 and anti-calretinin double labeled sections, a 1:200 dilution of affinity purified anti-TRPM3 and 1:20 dilution mouse monoclonal anti-calretinin (sc-365956; Santa Cruz Biotechnology) were used, followed by a 1:2000 dilution of anti-mouse Alexa Fluor 488 and anti-rabbit Alexa Fluor 594 secondary antibodies. Second, for the EAAT1 and LacZ double labeled sections (Fig. 1*B*), rabbit anti-EAAT1 (Abcam ab416) 1:500 dilution and chicken anti-β-galactosidase (β-Gal; Abcam 9361) 1:300 in 5% donkey serum, 0.5% Triton X-100, and 0.2% sodium azide in PBS were used as primary stain overnight at 4°C. Finally, rabbit anti-RBPMS 1:500 (Phospho Solutions, #1830) and chicken anti-β-Gal 1:300 were used for the RBPMS and LacZ double labeling, followed by anti-rabbit Alexa Fluor 594 and anti-chicken Alexa Fluor 488 secondary antibodies.

The TRPM3 and calretinin double labeled sections were imaged on a Leica TCS SP8 X confocal microscope using a 63×/1.40 NA oil immersion objective. Staining versus distance between the outer plexiform layer (OPL) and inner limiting membrane (ILM) was measured using the image-processing software, FIJI. Six 10 × 70–80 μm rectangular regions of interest (ROIs), oriented such that the length spanned the region of intensity between the OPL and ILM, were placed in the field randomly. The ROI Manager and Plot Profile functions were used to generate average fluorescence intensity values as a function of distance. Custom scripts in Python were used to normalize and graph the data. Sections for EAAT1/LacZ double label and RBPMS, LacZ double label were imaged on a Zeiss 780 AxioExaminer confocal microscope with either a 40×/1.4 NA oil immersion or 63×/1.4 NA oil immersion objective. Images were adjusted for brightness and contrast and pseudocolored using Leica software and FIJI (NIH ImageJ).

### Acutely isolated retinas

Animals were deeply anesthetized with isoflurane and decapitated. After enucleation of the eyes, retinas were dissected in oxygenated (95% O_2_/5% CO_2_) ACSF (119 mM NaCl, 2.5 mM KCl, 1.3 mM MgCl_2_, 1 mM K_2_HPO_4_, 26.2 mM NaHCO_3_, 11 mM D-glucose, and 2.5 mM CaCl_2_) at room temperature under infrared illumination. Isolated retinas were mounted RGC side up on nitrocellulose filter paper (Millipore), and transferred to a recording chamber of an upright microscope for imaging and simultaneous electrophysiological recording. The whole-mount retinas were continuously perfused (3 ml/min) with oxygenated ACSF at 32–34°C for the duration of the experiment.

### c-Fos staining experiment

P10 retinas were dissected as described above and maintained at 33C in an incubator in oxygenated ACSF; 50 μM PregS or vehicle control was added to the ACSF for 30 min followed by washout 3× with oxygenated ACSF, and then incubation in oxygenated ACSF for 1.5 h for all conditions. The retina pieces were then fixed for 30 min in 4% PFA in PBS, followed by wash in 0.1% PBST. To block mouse endogenous antibodies, we incubated overnight in F(ab) fragment anti-mouse IgG (H + L) 0.1 mg/ml (ab6668; Abcam; 1:10 dilution) and 1:1000 rat anti-mouse monoclonal CD16/CD32 antibody (UCSF MAB core) prepared in 0.1% PBST. Retina pieces were again washed 3× in PBST followed by a 4 h incubation in blocking buffer: 5% donkey serum, 0.2% sodium azide, 0.1% Triton X-100. Anti-c-Fos antibody [2H2] (ab208942) 1:1000 was incubated overnight in blocking buffer. Retina pieces were washed 3× and then incubated in goat anti-mouse Alexa Fluor 488 (1:1000) overnight. Retina pieces were briefly washed in water and then coverslipped using Fluoromount with DAPI and imaged on a Zeiss 780 confocal microscope with a 63× oil immersion objective (NA 1.4).

### Two-photon calcium imaging

Retinas were bolus loaded with Cal-520 AM (AAT Bioquest) using the multicell bolus loading technique (Stosiek et al., 2003) as previously described (Blankenship et al., 2009). Cal-520 AM was prepared at a concentration of 906.6 μm in 20% Pluronic in DMSO solution, which was then diluted 1:10 in ACSF, pH 7.4, sonicated, and filtered at 0.45 μm to remove particulates. Boroscilicate glass micropipettes (Sutter Instruments) were pulled to an approximate lumen of 1–2 μm (Narishige, PC-10) and used for pressure injection of the dye (World Precision Instruments, PV-820 Pneumatic PicoPump) at a pressure of 10–20 psi with the pipette positioned just under the ILM. Two-photon calcium imaging of neurons in the RGC layer was performed on a custom-built two-photon microscope with a 60× objective (Olympus 60×, 1 NA, LUMPlanFLN) and an ultrafast pulsed laser (Chameleon Ultra, Coherent) tuned to 920 nm. The microscope was controlled by ScanImage software (version 3.8; https://www.scanimage.org). Scan parameters were: 128 × 128 (6 Hz), or 256 × 256 (3 Hz), at 1 ms/line. Five- or 10-min imaging epochs were used and retinas were maintained at 33°C for the duration of the experiment.

### Calcium imaging analysis

Image stacks (movies) were imported into FIJI (Schindelin et al., 2012). Movies were spatially median filtered (0.5 × 0.5 pixels) to remove high-frequency noise and motion corrected using the NoRMCorre package (Pnevmatikakis and Giovannucci, 2017). ROIs were manually identified by selecting RGCs from average intensity projections. For each ROI, the baseline fluorescence (F_0_) was computed by taking the temporal median of all fluorescence values from the movie. Each fluorescence value (F) was normalized by dividing its difference from the baseline (F-F_0_) by the baseline [(F-F_0_)/F_0_] to produce a ΔF/F_0_ trace.

### Prolonged transient analysis

ΔF/F_0_ traces were median filtered in time (3-s window) to remove high-frequency noise. ΔF/F_0_ traces were Z-scored using the mean and SD calculated during the first 90 s of the movie, before bath application of the drug. Prolonged transients were defined as intervals during which Z-scored ΔF/F_0_ traces continuously exceeded 3 SD for at least 30 s. If a cell had multiple prolonged transients only the first was measured.

### Retinal wave analysis

Detection of retinal waves relied on finding and selecting their time scale relative to optical noise and mechanical drift of the tissue, along with detecting their highly synchronous activations of populations of neighboring neurons. ΔF/F_0_ traces were median filtered in time (3-s window) to remove high-frequency noise. Slow (minutes-long) fluctuations in F that arise from mechanical tissue drifts and bleaching were removed using the msbackadj function (Andrade and Manolakos, 2003). ΔF/F_0_ traces were then Z-scored. Cells were considered to be active if their Z-scored fluorescence exceeded 1.5 SD. Movie frames during which >10% of cells in the FOV were simultaneously active for at least 2 s were defined to depict a wave. Wave participation was defined as the proportion of cells that were active at any time during the wave. Wave frequency was defined as the number of waves divided by the length of the movie. Wave amplitude was defined as the mean of the distribution of cellular peak ΔF/F_0_ values during the wave. Wave duration was defined as the mean of the distribution of cellular full-width-at-half-maxima around their peak ΔF/F_0_ during the wave.

### Electrophysiological recordings

Whole-cell voltage clamp recordings were made from hemisectioned retinas continuously superfused in oxygenated ACSF (32–34°C) at a rate of 2–4 ml/min. Retinas were visualized under infrared illumination (870 nm). The ILM was removed using a glass recording pipette. Borosilicate glass recording pipettes (Sutter Instruments) were pulled (PP-830; Narishige) with tip resistance of 3–6 MΩ and filled with cesium gluconate internal solution containing the following: 110 mM CsMeSO_4_, 2.8 mM NaCl, 20 mM HEPES, 4 mM EGTA, 5 mM TEA-Cl, 4 mM ATP-Mg, 0.3 mM GTP-Na_3_, 10 mM phosphocreatine-Na_2_, and 5 mM QX-314, pH adjusted to 7.2 and with an osmolarity of 290 mOsm/kg H_2_O. The liquid junction potential correction for this solution was −13 mV. Voltage-clamp recordings were obtained from somas of RGCs (holding potential of −60 mV) as 10-min gap free recording using pCLAMP10 recording software and a Multiclamp 700A amplifier (Molecular Devices), sampled at 20 kHz and low-pass filtered at 2 kHz. For current clamp experiments, internal solution contained the following: 116 mM K^+^ D-gluconate, 6 mM KCl, 2 mM NaCl, 20 mM HEPES, 0.5 mM EGTA, 4 mM ATP-Mg^2+^, 0.3 mM GTP-Na_3_, and 10 mM phosphocreatine-Na_2_.

### Pharmacology

**Figure 2.**
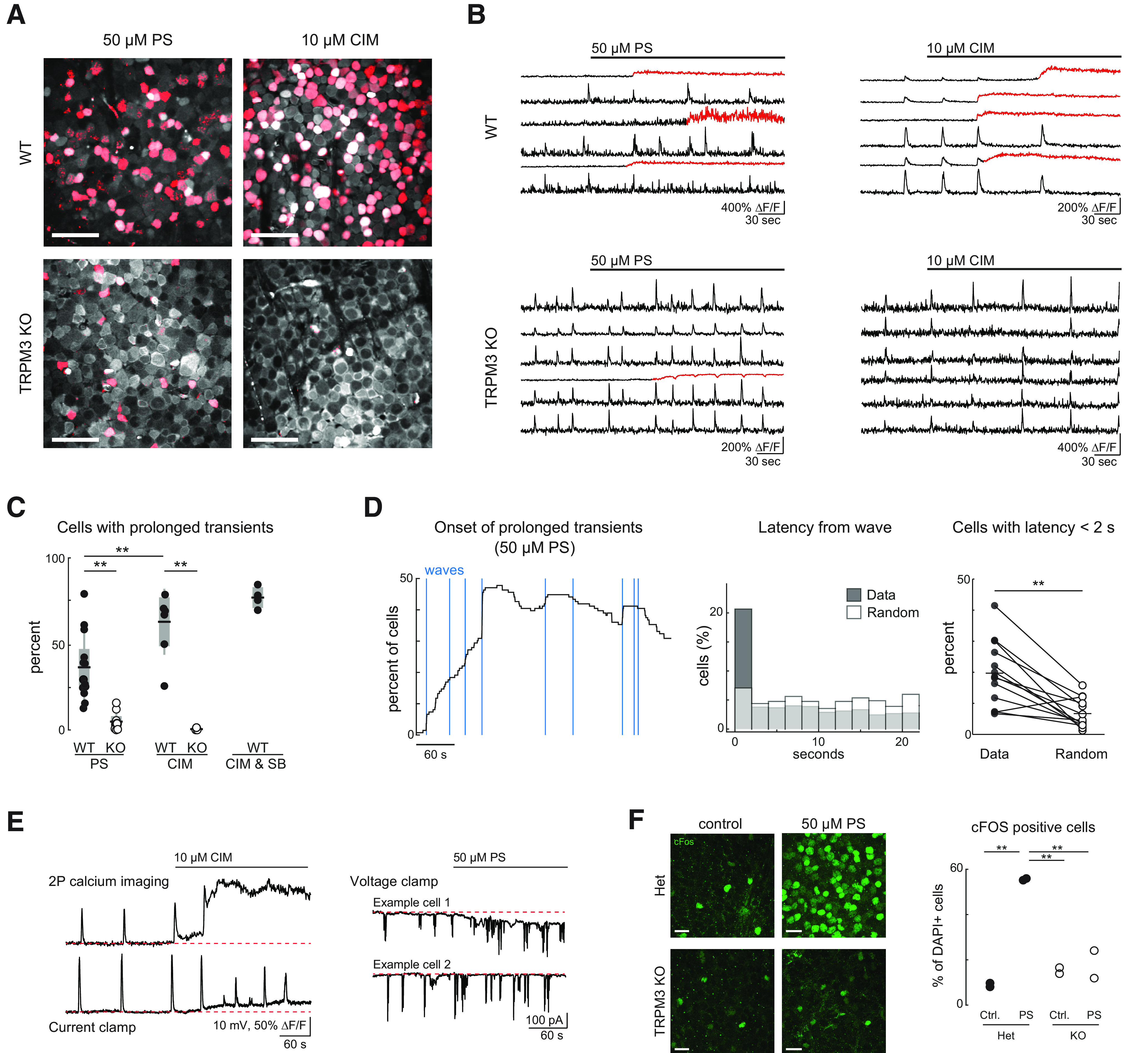
Activation of TRPM3 induced prolonged calcium transients in a subset of RGCs. ***A***, Maximum intensity projection of the RGC layer after loading with Cal-520 AM calcium indicator. Red overlay, Cells that exhibited prolonged calcium transients during drug application. Scale bars: 50 μm. ***B***, Representative ΔF/F traces. Red portions, Prolonged calcium transients. ***C***, Comparison of proportion of cells that exhibited prolonged calcium transients. SB = synaptic blockers (50 μM D-AP5, 20 μM DNQX, 8 μM DHβE, 4 μM strychnine, and 5 μM gabazine); *p* values are from Tukey–Kramer *post hoc* tests following a two-way ANOVA (there was a significant interaction between drug and genotype: *F* = 6, *p* = 0.021); *N* = 13 experiments from 7 mice (WT, PS); *N* = 10 experiments from 6 mice (KO PS); *N* = 8 experiments from 4 mice (WT CIM); *N* = 3 experiments from 2 mice (KO CIM); and *N* = 4 experiments from 2 mice (WT, CIM+ SB). For all figures, *p < 0.05 and **p < 0.005. ***D***, Onset of prolonged transients are correlated with waves. Left, Proportion of cells exhibiting a prolonged transient throughout an example movie. Note that many cells initiate prolonged transients at the onsets of waves (blue lines). Middle, Latencies between prolonged transient initiations and the most recent wave drawn from real initiation times compared with latencies of between randomized initiation times drawn from a uniform distribution and the most recent wave; *N* = 900 cells from 13 experiments on 7 mice. Right, Proportion of cells with a prolonged transient initiation <2 s after the initiation of a wave compared, for each experiment, between real and randomized prolonged transient initiation times. Same dataset as ***E***. ***E***, left, Example simultaneous current clamp recording from RGC and two-photon calcium imaging in ganglion cell layer during bath application of TRPM3 agonist CIM. In this cell, there was a small tonic depolarization associated with bath application of CIM. Right, Two example voltage clamp recordings from RGCs during bath application of PregS. In example cell 1, there is a tonic inward current associated with PregS, while in example cell 2, there was no change in holding current. Summary data provided in main text. ***F***, Prolonged calcium transients correlated with expression of cFOS. Left, Representative images of immunohistochemistry staining against the immediate early gene product c-Fos (green). Images are maximum intensity projections through the ganglion cell layer of TRPM3 Het or KO retinas exposed to 50 μm PregS. Scale bar: 20 μm. Right, Proportion of c-Fos+ cells relative to the total number of DAPI+ cells (*n* = 2 retinas from 2 mice per experimental group; *n* = 293 cells for WT Ctrl., *n* = 322 cells for WT PS, *n* = 289 cells for KO Ctrl., and *n* = 330 cells for KO PS). ANOVA with Tukey HSD, **p < 0.01, *p < 0.05.

For pharmacology experiments, imaging and/or electrophysiology recording was conducted under baseline conditions for at least 10 min before application of pharmacological agents. Pharmacological agents were added to oxygenated ACSF, maintained at 33°C, and bath perfused onto the retina after 1–2.5 min following the start of imaging or recording to obtain both stable baseline and change due to the agent in the same imaging/recording epoch. Pharmacological agents were used in the following concentrations: PregS 50 μM (Sigma-Aldrich) and 10–50 μM CIM (Tocris). To block all fast synaptic transmission (Fig. 2*C*), we used 50 μM D-AP5, 20 μM DNQX, 8 μM DHβE, 4 μM strychnine, and 5 μM gabazine. All compounds were purchased from Tocris Bioscience and prepared in dH_2_O and diluted to the final concentration in ACSF.

## Results

### TRPM3 in the developing retina is localized to inner retinal processes and RGCs

In the adult retina, TRPM3 is localized to processes in the inner plexiform layer (IPL), a subset of RGC somas, and potentially other cells including displaced amacrine cells (Brown et al., 2015). TRPM3 mRNA and TRPM3 promoter-driven expression of a reporter have been demonstrated in Müller glia (Gilliam and Wensel, 2011; Hughes et al., 2012). To characterize the localization of TRPM3 during development, we used two approaches. First, we used a custom antibody targeted against the C terminus of mouse TRPM3 (Brown et al., 2015). To demarcate synaptic layers in the developing IPL we co-stained for calretinin, a calcium binding protein localized to a subset of amacrine and ganglion cells whose processes define IPL sublamina after eye-opening (Fig. 1). From P9 to P15, we observed punctate TRPM3 immunofluorescence throughout the IPL as well as labeling of RGCs, many of which were also positive for calretinin (Fig. 1*A*). Faint labeling was also seen over some inner retinal neurons. TRPM3 immunofluorescence increased between P9 and P15, with the labeling at P15 closely resembling that of the adult (Brown et al., 2015).

Second, we used a TRPM3 +/− mouse, which contains β-Gal in place of the TRPM3 coding sequence (Hughes et al., 2012; Fig. 1*B*). Similar to studies in the adult retina, we observed high levels of β-Gal in the IPL and patterns of expression that flanked RGCs, appearing consistent with Müller glial end feet and lateral processes. We also observed lower levels of β-Gal expression in the RGCs themselves. We observed co-localization of β-Gal with EAAT1 immunofluorescence, which labels a glial glutamate transporter, consistent with strong expression in Müller glial cells. In addition, somatic staining for β-Gal in the GCL co-localized with the RGC marker RBPMS. Together, these data indicate that, during development, TRPM3 is expressed in developing RGCs, Müller cells, and potentially other inner nuclear layer (INL) neurons.

### Activation of TRPM3 induced prolonged calcium transients and expression of the immediate early gene c-Fos in a subset of RGCs

To determine whether TRPM3 channels are functional in the developing retina, we bath applied TRPM3 agonists while imaging calcium responses within the ganglion cell layer of P8-P12 retinas loaded with the chemical calcium indicator Cal-520 AM (Fig. 2). Addition of the neurosteroid PregS (50 μM) induced large and prolonged calcium transients in a subset of RGCs (36.4 ± 19.6% cells undergoing prolonged transients, *n* = 13 experiments from 7 mice; Fig. 2*A–C*). RGCs with large transients were evenly distributed throughout the field of view (Fig. 2*A*).

We repeated these experiments in TRPM3 KO retinas and found that PregS induced prolonged calcium transients in a significantly lower percentage of RGCs (4.8 ± 5.2% cells; *p* = 0.0002; *n* = 10 experiments from 6 mice; Fig. 2*A–C*). Although the identity of the remaining cells that exhibited prolonged calcium transients in TRPM3 KO is not known, it is worth noting that they formed a near-mosaic distribution and their somas had similar morphologies, suggesting they comprised a particular subtype of retinal neuron (Fig. 2*A*). Note that RGCs still exhibited wave-associated calcium transients in TRPM3 KO retinas, as described below. Together, these data indicate that the majority of PregS-induced prolonged transients occurred via activation of TRPM3 channels, while a small subset of prolonged transients occurred through a mechanism independent of TRPM3.

Bath application of the TRPM3 synthetic ligand CIM in WT retina induced similar prolonged transients, however in a significantly higher percentage of cells when compared with PregS (62.9 ± 19.2% cells in CIM; *p* = 0.0058; *n* = 8 experiments from 4 mice; Fig. 2*C*). This effect was absent in TRPM3 KO mice (0.4 ± 0.8% of cells exhibit prolonged transients in KO; *p* < 0.0001; *n* = 3 experiments from 2 mice). CIM is highly selective for TRPM3, but is thought to activate a different conductance state (Held et al., 2015a), which could potentially explain the stronger effect on RGCs. CIM-induced prolonged transients persisted in the presence of blockers of fast neurotransmitters (*n* = 4 experiments from 2 mice), indicating it was acting directly on RGCs.

We noted that PregS-induced prolonged transients were not synchronized across the field of view. Rather in a subset of cells, the prolonged transients occurred simultaneous with waves, indicating a synergistic interaction (Fig. 2*D*). Indeed, we find that a significantly larger proportion of cells exhibit prolonged transients within 2 s of a wave than would be expected for random occurrences (19.7 ± 10.4% of cells with sub-2-s initiation latency following wave in experimental data; 6.7 ± 4.7% of cells in shuffled data; *p* = 0.0004; *n* = 13 experiments from 7 mice). Together, these data seem to indicate that the depolarization provided by retinal waves contributes to, but is not required for, the induction of prolonged calcium transients by TRPM3 agonists.

To begin to understand the origin of these prolonged calcium transients, in a subset of imaging experiments we also conducted simultaneous whole-cell current clamp or voltage clamp experiments during the application of either PregS or CIM. In voltage clamp experiments, 5 out of 9 cells exhibited tonic inward current ranging from 5 to 20 pA with one cell exhibiting a substantial tonic inward current of 60 pA. In current clamp experiments, five out of eight cells exhibited tonic depolarization ranging from 3 to 20 mV that was not associated with bursts of action potentials (Fig. 2*E*). Note that this small impact on membrane depolarization indicates that the bulk of the calcium increase is not due to direct influx via voltage-gated calcium channels. Rather the data are consistent with activation of TRPM3 leading to a release from internal stores. The various potential sources of calcium underlying the prolonged transients are discussed below.

Large increases in intracellular calcium concentration associated with TRPM3 channels have been observed in several cell types and have been associated with activation of several pathways that influence gene expression (Thiel et al., 2013; Rubil et al., 2016). To test whether PregS-induced prolonged transients were associated with changes in gene expression in RGCs, we stained for c-Fos in TRPM3 heterozygous and KO retinas exposed to PregS or control treatment for 30 min before fixation. Consistent with our calcium imaging results, we observed a larger proportion of c-Fos/DAPI-positive cells in the GCL of PregS-treated heterozygous retinas than in PregS-treated TRPM3 KO retinas (55.6 ± 0.5% heterozygous cells expressing c-Fos after PregS treatment, 18.0 ± 8.6% TRPM3 KO cells expressing c-Fos after PregS; *p* = 0.0022; *n* = 2 retinas from 2 mice per experimental group; Fig. 2*F*).

### PregS increases the frequency of spontaneous synaptic events in RGCs via a TRPM3-dependent mechanism

**Figure 3.**
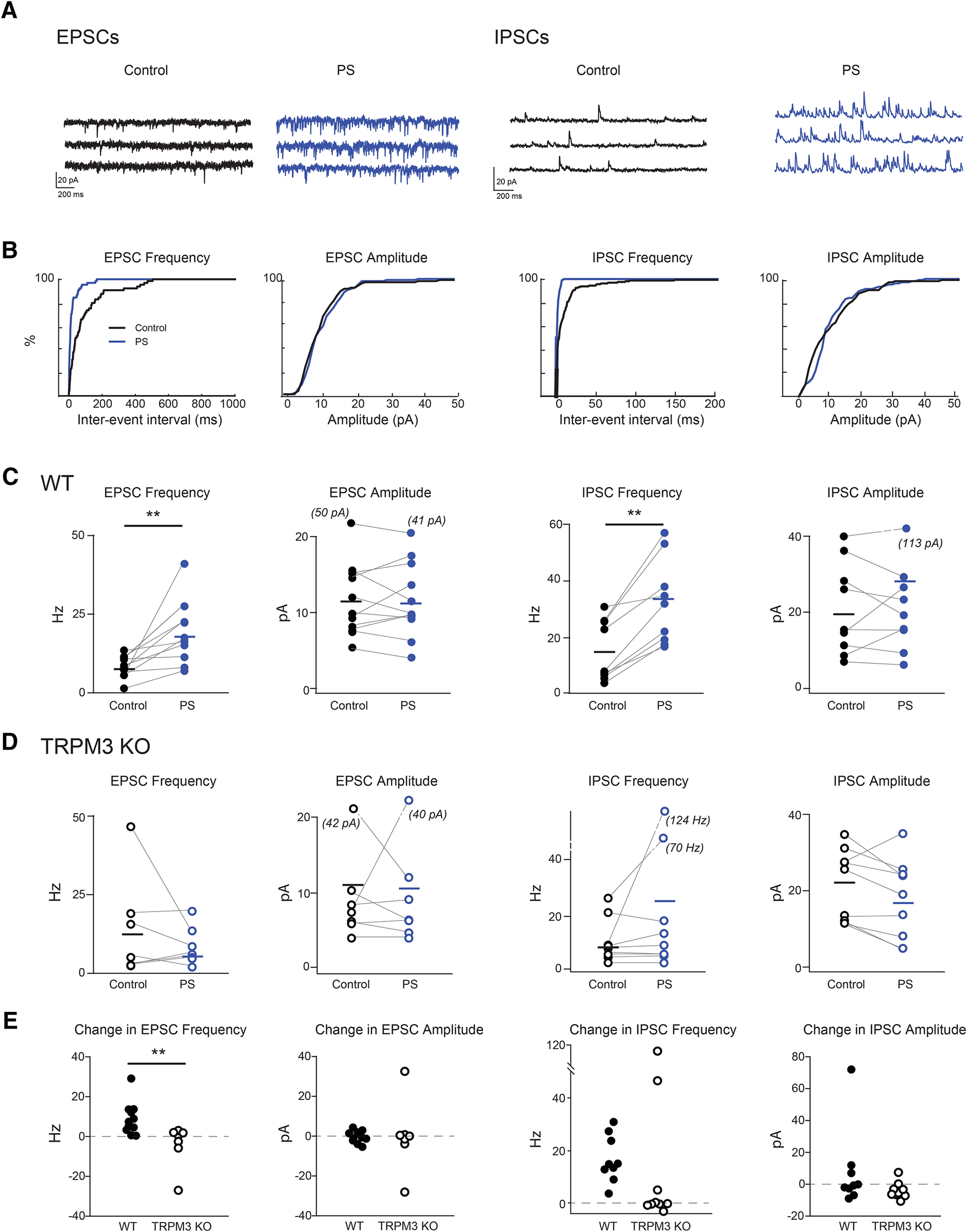
Addition of PregS increased the frequency but not amplitude of sEPSCs and sIPSCs onto RGCs. ***A***, Example spontaneous PSCs recorded from a RGC in control conditions and in the presence of 50 μm PregS. Left, Cell is held at −60 mV to isolate sEPSCs. Right, Cell is held at 0 mV to isolate sIPSCs. Black = control conditions, blue = PregS. ***B***, Cumulative probability distribution of interevent interval and amplitude of sEPSCs (left) and sIPSCs (right). Black = control conditions, blue = PregS. ***C***, Summary of the effects of PregS on frequency and amplitude of PSCs (*n* = 9 RGCs in each condition from 8 mice for EPSCs and 7 mice for IPSCs), line = average. ANOVA with Tukey HSD, ** *p* < 0.01, * *p* < 0.05. Values for outliers shown in parentheses. ***D***, Same as ***C*** for PSCs recorded in RGCs in TRPM3 KO mice (*n* = 6 RGCs in each condition, 3 mice for EPSCs and 5 mice for IPSCs). ***E***, Summary of fold change of PregS on PSC amplitude and frequency comparing WT and TRPM3 KO mice. ANOVA with Tukey HSD, **p < 0.01, *p < 0.05.

Inspired by studies in the developing cerebellum where activation of TRPM3 increases the frequency of spontaneous synaptic events recorded in Purkinje cells (Zamudio-Bulcock and Valenzuela, 2011), we performed whole-cell voltage clamp recordings from RGCs wherein we compared the frequency and amplitude of spontaneous synaptic events. Addition of PregS increased the frequency (control: 6.04 ± 2.73 Hz, PregS: 15.04 ± 8.21 Hz; *p* = 0.001), but not the amplitude of spontaneous EPSCs (sEPSCs; control: 14.20 ± 12.34 pA; PregS: 13.92 ± 10.91 pA; *p* = 0.96; *n* = 11 cells from 8 mice; Fig. 3*A–C*). A similar increase in frequency (control: 15.33 ± 10.76 Hz, PregS: 32.22 ± 15.09 Hz; *p* = 0.0039) but not amplitude (control: 20.91 ± 12.35 pA, PregS: 28.62 ± 32.53 pA; *p* = 1; *n* = 9 cells from 7 mice) following addition of PregS was observed among spontaneous IPSCs (sIPSCs; Fig. 3*A–C*). This PregS-induced increase in frequency of spontaneous synaptic events was not observed in TRPM3 KO retinas (WT vs KO change in EPSC frequency *p* = 0.0041; *n* = 7 cells in 3 KO mice; WT vs KO change in IPSC frequency *p* = 0.0625; *n* = 9 cells in 5 KO mice; Fig. 3*D*,*E*). Taken together, the electrophysiology data point to a presynaptic mechanism of action of TRPM3. Hence, TRPM3 alters neurotransmitter release properties presynaptic to RGCs.

### PregS modulates retinal waves via a TRPM3-independent mechanism

As the retina develops, retinal waves are mediated by different retinal circuits (Blankenship and Feller, 2010). Given the strong expression of TRPM3 during the second postnatal week (Fig. 1) and its impact on glutamatergic synapses (Fig. 3), we focused on retinal activity during glutamatergic waves, which occur between P11 and P15.

**Figure 4.**
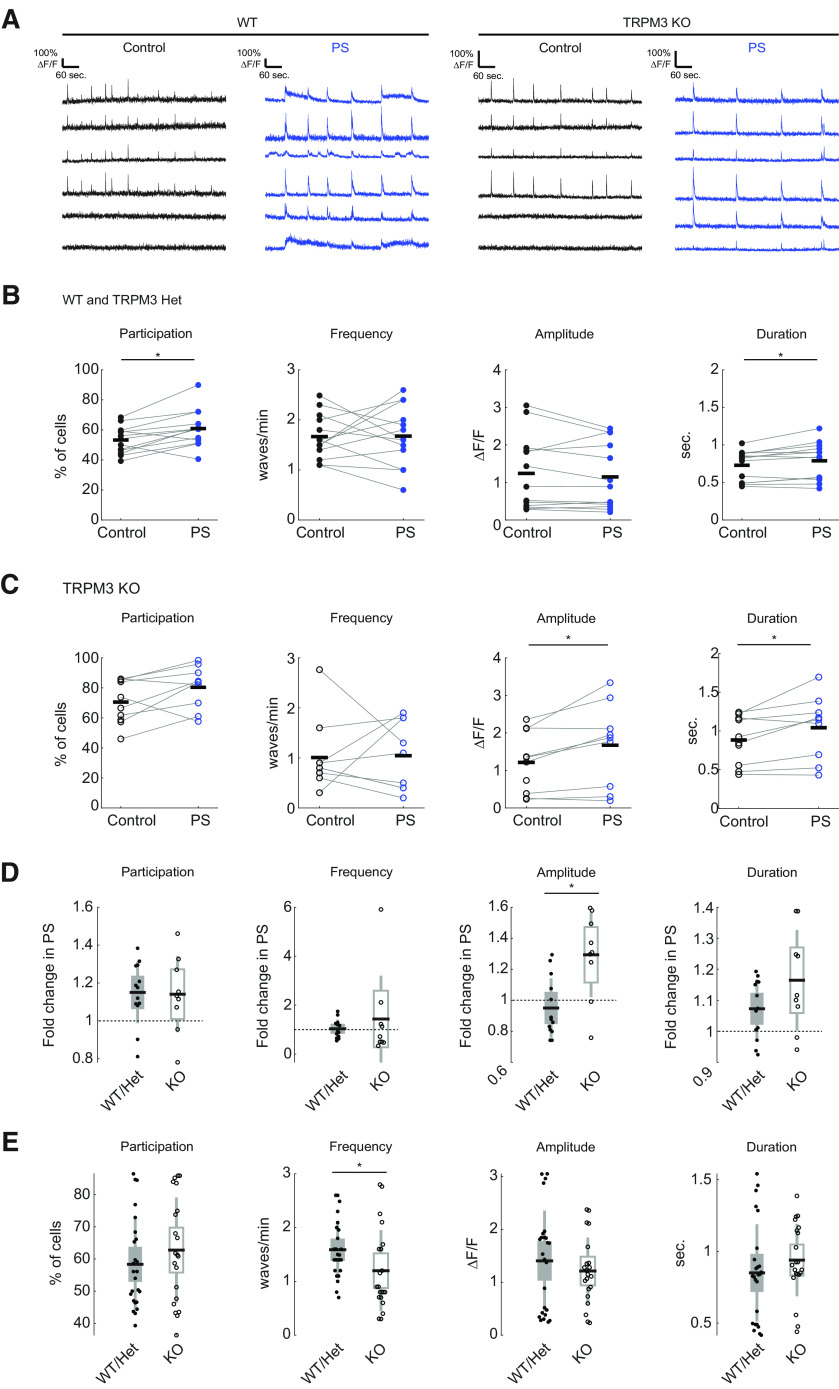
PregS modulates retinal waves via a TRPM3-independent mechanism. ***A***, Representative ΔF/F traces. ***B***, Comparison of wave properties before and after bath application of 50 μM PregS (PS) in WT and TRPM3 heterozygous (Het) mice (*n* = 13 experiments in 7 mice; 6 WT and 1 Het); *p* values in ***B***, ***C*** are from paired *t* tests. ***C***, Same as ***B*** but for TRPM3 KO mice (*n* = 10 experiments in 5 mice). ***D***, Comparison between genotypes of the extent of modulation of wave properties by bath application of PregS. Same dataset as ***B***, ***C***. ***E***, Comparison of wave properties between genotypes (*n* = 25 experiments in 10 mice, 9 WT and 1 Het; *n* = 21 experiments in 7 KO mice).

We monitored the effect of PregS on several features of retinal waves (Fig. 4). Addition of PregS did not significantly change the frequency of retinal waves or the amplitude of the calcium transients per wave (control frequency: 1.67 ± 0.45 waves/min, PregS frequency: 1.68 ± 0.60 waves/min, *p* = 0.9566; control amplitude: 1.24 ± 1.00 ΔF/F, PregS amplitude 1.15 ± 0.88 ΔF/F, *p* = 0.2850). PregS increased the total percent of RGCs that participate in waves as well as the duration of wave-associated calcium transients in WT retinas (control participation 53.3 ± 9.0%, PregS participation 60.9 ± 12.3%, *p* = 0.0065; control duration 0.73 ± 0.20 s, PregS duration 0.79 ± 0.25 s, *p* = 0.015; *n* = 13 experiments from 7 mice; Fig. 4*B*). We observed a similar effect of PregS on retinal waves in TRPM3 KO mice (control participation 70.5 ± 14.5%, PregS participation 80.3 ± 14.5%, *p* = 0.0748; control duration 0.88 ± 0.31 s, PregS duration 1.04 ± 0.41 s, *p* = 0.0272; *n* = 10 experiments from 5 mice; Fig. 4*C*,*D*). Interestingly, we also observed a PregS-induced increase in wave-associated calcium transient amplitude in KO retinas that was absent in WT/Het retinas, suggesting a modulatory role for non-TRPM3 targets in retinal waves that is revealed in TRPM3 KO (WT/Het amplitude fold change in PregS 1.0 ± 0.2, KO amplitude fold change 1.3 ± 0.3, *p* = 0.0076; *n* = 13 experiments from 7 mice; Fig. 4*D*).

To test whether endogenous activation of TRPM3 modulated retinal waves, we compared wave properties between WT/Het and TRPM3 KO mice. We found that the absence of TRPM3 had minimal impact on the amplitude (WT/Het 1.4 ± 1.0 ΔF/F, KO 1.2 ± 0.6 ΔF/F, *p* = 0.4942), duration (WT/Het 0.8 ± 0.3 s, KO 0.9 + 0.3 s, *p* = 0.1786), and cell participation in retinal waves (WT/Het 58.4 ± 14.0%, KO 62.8 ± 16.3%, *p* = 0.3777), although waves occurred at a slightly lower frequency in TRPM3 KO retinas (WT/Het frequency 1.6 ± 0.5 waves/min, KO frequency 1.2 ± 0.8 waves/min, *p* = 0.0296; *n* = 25 experiments from 10 WT/Het mice; *n* = 21 experiments from 7 KO mice; Fig. 4*E*). Together, these data indicate that PregS is primarily exerting its influence on wave generating circuits independent of TRPM3 channels, and that TRPM3 is not necessary for wave generation and propagation. However, the effect of genotype on baseline wave frequency and on PregS-associated change in amplitude suggest that TRPM3 plays a modulatory role in regulating spontaneous activity during development.

## Discussion

We have demonstrated that the multimodally regulated ion channel TRPM3 is present, is functional, and modulates spontaneous synaptic activity in the developing retina. First, we showed that TRPM3 is present in RGCs during development and provide evidence for additional expression in Müller glia. Second, using two-photon calcium imaging, we show that application of the TRPM3 agonist PregS causes a prolonged increase in intracellular calcium concentration that is triggered by retinal waves, a phenomenon that is nearly absent in the TRPM3 KO and mimicked by the TRPM3 synthetic agonist, CIM. Using whole-cell voltage clamp recordings from RGCs, we found that application of the neurosteroid PregS increased the frequency of sEPSCs and sIPSCs, again an effect absent in TRPM3 KO, indicating that activation of TRPM3 alters signaling presynaptic to RGCs. Finally, we found that PregS modulates spontaneous retinal wave activity during a critical time of development via both TRPM3-dependent and -independent mechanisms. These data provide insight into the roles of this unconventional TRPM3-dependent and TRPM3-independent PregS signaling and the role of TRPM3 in regulating spontaneous activity in early retinal development.

### TRPM3 impact on RGCs

In the adult retina, TRPM3 is expressed in a distinct subset of RGCs as determined by antibody staining (Brown et al., 2015), reporter expression (Hughes et al., 2012), and functional assays on dissociated RGCs (Brown et al., 2015). Interestingly, TRPM3 KO mice exhibit an altered pupillary light reflex (Hughes et al., 2012), a light-evoked pupil constriction mediated by intrinsically photosensitive RGCs integrating rod and cone-originating synaptic input with intrinsic melanopsin phototransduction. Although the pupil constriction in response to light stimulation is rapid and maintained throughout the exposure to light, the pupils did not reach a full constriction and had a significantly attenuated post-stimulus response, both phenotypes that are reminiscent of mice lacking melanopsin. A TRPM3 promoter-driven reporter was not found to co-localize with melanopsin containing intrinsically photosensitive RGCS (ipRGCs; Hughes et al., 2012), suggesting that its impact on the pupillary light reflex may be indirect modulation via activation of glial cells or cells within the ciliary body. However, TRPM3 mRNA has been shown to be expressed in ipRGCs, demonstrating a possibility that TRPM3 may be directly activated in ipRGCs (Rheaume et al., 2018).

We provide several lines of evidence indicating that TRPM3 is present in RGCs during development. First, antibody staining indicated that a subpopulation of cells in the ganglion cell layer express TRPM3, similar to the pattern in the adult. Second, visualization of cells in TRPM3+/− mice, in which a β-Gal cassette replaces the coding sequence of TRPM3 (Hughes et al., 2012), replicated the staining pattern in the GCL that we observed with the antibody. Third, application of the synthetic TRPM3 agonist, CIM, caused prolonged calcium transients associated with retinal waves, an effect absent in the TRPM3 KO.

What is the source of the calcium for the prolonged transients? Activation of TRPM3 by PregS and/or CIM causes large increases in intracellular calcium in a variety of cells including pancreatic islet cells (Wagner et al., 2008; Held et al., 2015a), smooth muscle cells (Naylor et al., 2010), and isolated RGCs (Brown et al., 2015). TRPM3 induced calcium increases are likely triggered by calcium influx via the TRPM3 channel itself, although the calcium permeability of the channel depends on which splice isoform is expressed (Grimm et al., 2003). Given the sustained nature of the calcium influx in different cell types and what we observe in RGCs (Fig. 2*A*), the bulk of the calcium is likely to be release from intracellular stores, potentially via calcium-induced calcium release. Indeed, TRPM3-mediated increases in intracellular calcium are tied to specific signaling pathways, including those that induce activation of c-Fos (Thiel et al., 2013).

We observed an interesting synergism between activation of TRPM3 via PregS and retinal waves. Bath application of PregS did not cause a synchronous depolarization of all RGCs, but rather appeared to cause a prolonged transient when a wave co-occurred, with different cells being affected by different waves. We speculate that this synergism may be due to the multiple sites of activation/modulation of TRPM3 channels (Held et al., 2015b; Thiel et al., 2017). For example, waves provide a strong depolarization and/or an influx of calcium that could facilitate the opening of the channel in the presence of PregS. Insights into the molecular mechanisms underlying the source of this synergism would require a deeper understanding of the channel. However, these data indicate that the presence of neuronal hormones can have a dramatic effect on the amplitude and duration of calcium transients that follow depolarizations evoked by spontaneous network activity in developing neural circuits.

### TRPM3 affects retinal signaling via presynaptic mechanisms and/or glial signaling

In the adult retina, TRPM3 is not confined to RGCs, instead, there is also extensive immunolabeling in the inner nuclear layer (Brown et al., 2015), as well as expression in Müller glia (Gilliam and Wensel, 2011). We found a similar staining pattern in the developing retina. Moreover, we demonstrated that the TRPM3 reporter, β-Gal, is present in the same cells as EAAT1, a marker for Müller glial cells, indicating strong expression in Müller cells during development.

The function of INL/glial TRPM3 in the adult retina is not known. TRPM3 KO mice have normal ERGs, indicating normal function of the outer retina (Brown et al., 2015). During development, we observed that the TRPM3 agonist PregS increased the frequency of sIPSCs and sEPSCs recorded from RGCs, suggesting there was an increase in excitability presynaptic to RGCs. A similar TRPM3-associated increase in EPSC frequency was observed in the developing cerebellum (Zamudio-Bulcock and Valenzuela, 2011) and the adult hippocampus (Mameli et al., 2005) consistent with a presynaptic site of action. Another potential mode of action of TRPM3 agonists in Müller glia is an increased excitability via changes in the function of glial transporters that could lead to elevation of excitatory substances, such as extracellular potassium (Khakh and Sofroniew, 2015). Whether the increased IPSC/EPSC frequency in retina occurs directly via activation of bipolar cells or indirectly via Müller glia remains to be determined.

A third possibility is that the strong depolarization of some RGCs is facilitating a retrograde influence on presynaptic release by a diffuse messenger, such as NO or reactive oxygen species. Such retrograde signaling has been implicated in retinal development (Du et al., 2009; Johnson and Kerschensteiner, 2014).

### Implication for PregS and TRPM3 signaling in neural circuits and development

PregS has been implicated in a variety of physiological roles in the adult hippocampus. For example, PregS has been shown to modulate hippocampal plasticity, increasing synaptic activity in an age-dependent (Mameli et al., 2005), and stress-dependent manner (Flood et al., 1992; Barbaccia et al., 1996). In other studies, intracranial PregS injection has been shown to increase the survival of newborn neurons in the dentate gyrus (Mayo et al., 2005; Yang et al., 2011). Although it is not yet known whether these PregS dependent phenomena occur via TRPM3 or other receptors, a truncation mutation of TRPM3 is associated with autism spectrum disorder when coupled to a mutation of the *dmd* gene, frequently associated with muscular dystrophy (Pagnamenta et al., 2011). Moreover, PregS has the highest expression of all hormones in amniotic fluid (Bicíková et al., 2002) and umbilical cord blood concentrations spike during gestation (Adamcová et al., 2018). We speculate that this high dose of PregS could influence activity in developing circuits and potentially induce cellular changes during development via altered gene expression, which we observed happens in a TRPM3 dependent fashion. Interestingly, PregS has been shown to have cognitive enhancing properties in clinical trials for schizophrenia (Marx et al., 2011) and autism spectrum disorder (Fung et al., 2014); however, the mechanism of action was generally proposed to be as a positive allosteric modulation of NMDA receptors. Given the TRPM3-dependent synaptic and gene regulation activity of PregS that we report, it may be fruitful to further investigate TRPM3 as a possible therapeutic target in developmental CNS disorders.

## Synthesis

Reviewing Editor: Douglas Bayliss, University of Virginia School of Medicine Decisions are customarily a result of the Reviewing Editor and the peer reviewers coming together and discussing their recommendations until a consensus is reached. When revisions are invited, a fact-based synthesis statement explaining their decision and outlining what is needed to prepare a revision will be listed below. The following reviewer(s) agreed to reveal their identity: R. Lane Brown, Joshua Singer.

Synthesis

This manuscript was seen by two reviewers, who agree that the additional work with the Trpm3 knockout mice has greatly improved the paper. Whereas both note that many mechanistic issues remain unresolved, they agree that identifying Trpm3-dependent and -independent roles for PregS in modulating retinal activity waves is an interesting and novel observation.

In addition to addressing a number of minor points (see below), it was recommended that the data presented for the reviewers (in the rebuttal) should be strengthened and included in the manuscript proper. The main point in showing the “reviewer data” would be to provide evidence that the development of inward current results from TRPM3 expression in RGCs. It was felt that much of these data may be already available, and could be buttressed with recordings from knockout mice or using the CIM compound (which seems to have fewer off-target effects.)

---

Reviewer #1

Advances the Field

This manuscript describes functional roles of TRPM3 channels in the retina.

Comments to the Authors

I appreciate the effort of the authors to revise this manuscript and to perform new experiments on TRPM3 knock-out animals to clarify TRPM3-dependent and -independent effects of Pregnenolone Sulfate on retinal signaling. The effects of PS on PSCs are particularly interesting and novel. I would also question if the figure provided to the reviewers demonstrating the development of a PS-dependent current in a whole-cell recording should be reported in the manuscript. Although many questions remain, the manuscript is now greatly improved, but still requires some minor revision, as described below:

Line 53 - Authors mention “light detection in periphery”. It is unclear what is meant by this statement as the retinal is part of the central nervous system.

Line 69 - There are likely to be other effects on the retina. A better statement would be “in retinal ganglion cells”, although many effects, such as PS block of voltage-gated sodium channels, are likely invisible in this assay.

Line 81 - The P in P8 is redundant.

Line 117 - The mention of a “function of time” appears to be a typographical error, as the authors are measuring a function of distance.

Line 144 - “Fluoramount” is misspelled.

Line 149 - “AM” is missing

Line 215 - Delete mention of mefanamic acid, as this is no longer used in the experiments.

Line 261 - The authors should discuss if calcium transients are still associated with retinal waves in the TRPM3 knock-out animal.

Line 401 - It is unclear how a cytoplasmic protein such as beta-gal is “co-localized” with a membrane protein such as EAAT-1. The authors should use more precise language.

Reviewer #2

Advances the Field

No mechanistic insight into retinal wave generation or propagation; minimal mechanistic insight into retinal circuit development; minimal insight into TRPM3 function. It may be of interest that the paper suggests steroid signaling as relevant to the development of the retinal circuit.

Comments to the Authors

I appreciate that the authors have done some additional experiments to improve the revised study. The inclusion of data from experiments using TRPM3 knockouts is a strength. The absence of any rigorous electrophysiological analysis of the PregS effect remains a weakness: the data submitted in the rebuttal are not convincing, and I don’t understand why the authors couldn’t do current-clamp recordings with an internal solution that includes a fluorescent [Ca2+] indicator.

The study, in my opinion, remains of minimal significance given that it presents a single, unexplained and unexplored finding: PregS, acting in part on TRPM3 channels, modulates Ca2+ signaling in some retinal ganglion cells through an unknown mechanism.
